# Analyzing Monthly Blood Test Data to Forecast 30-Day Hospital Readmissions among Maintenance Hemodialysis Patients

**DOI:** 10.3390/jcm13082283

**Published:** 2024-04-15

**Authors:** Cheng-Han Tsai, Dong-Her Shih, Jue-Hong Tu, Ting-Wei Wu, Ming-Guei Tsai, Ming-Hung Shih

**Affiliations:** 1 Department of Information Management and Institute of Healthcare Information Management, National Chung Cheng University, Chiayi City 62102, Taiwan or 6675b@vghtc.gov.tw; 2 Department of Emergency Medicine, Chiayi Branch, Taichung Veteran’s General Hospital, Chiayi City 60090, Taiwan; 3 Department of Information Management, National Yunlin University of Science and Technology, Douliu 64002, Taiwan; wutingw@yuntech.edu.tw; 4 Department of Nephrology, St. Joseph’s Hospital, Yunlin 63241, Taiwan; 0001540@mail.stjoho.org.tw (J.-H.T.); hr@mail.stjoho.org.tw (M.-G.T.); 5 Department of Electrical and Computer Engineering, Iowa State University, 2520 Osborn Drive, Ames, IA 50011, USA; mshih@iastate.edu

**Keywords:** hemodialysis, machine learning, ensemble learning, hospital readmissions prediction

## Abstract

**Background**: The increase in the global population of hemodialysis patients is linked to aging demographics and the prevalence of conditions such as arterial hypertension and diabetes mellitus. While previous research in hemodialysis has mainly focused on mortality predictions, there is a gap in studies targeting short-term hospitalization predictions using detailed, monthly blood test data. **Methods:** This study employs advanced data preprocessing and machine learning techniques to predict hospitalizations within a 30-day period among hemodialysis patients. Initial steps include employing K-Nearest Neighbor (KNN) imputation to address missing data and using the Synthesized Minority Oversampling Technique (SMOTE) to ensure data balance. The study then applies a Support Vector Machine (SVM) algorithm for the predictive analysis, with an additional enhancement through ensemble learning techniques, in order to improve prediction accuracy. **Results:** The application of SVM in predicting hospitalizations within a 30-day period among hemodialysis patients resulted in an impressive accuracy rate of 93%. This accuracy rate further improved to 96% upon incorporating ensemble learning methods, demonstrating the efficacy of the chosen machine learning approach in this context. **Conclusions:** This study highlights the potential of utilizing machine learning to predict hospital readmissions within a 30-day period among hemodialysis patients based on monthly blood test data. It represents a significant leap towards precision medicine and personalized healthcare for this patient group, suggesting a paradigm shift in patient care through the proactive identification of hospitalization risks.

## 1. Introduction

The population of hemodialysis patients has been growing worldwide, particularly in low- and middle-income countries (LMICs), due to factors such as the increased availability of dialysis, aging populations, and the rising prevalence of conditions like arterial hypertension (AH) and diabetes mellitus (DM). Despite the expansion of dialysis services, globally, many individuals lack access to kidney replacement therapy (KRT), resulting in millions of deaths from kidney failure annually. Among those with access to dialysis, mortality rates remain high, underscoring the need for innovation to improve accessibility and patient outcomes [1].

Mortality among hemodialysis patients is notably high, especially within the first few months of initiating treatment. In high-income countries (HICs), about one-quarter of hemodialysis patients die within the first year of treatment, and the rates are even higher in LMICs. Over the past two decades, there have been improvements in survival rates for hemodialysis patients, with some data suggesting that younger patients have seen relative gains in survival. Comparatively, while short-term mortality has historically been lower for patients treated with peritoneal dialysis (PD) than those on hemodialysis, long-term risks were higher [2]. One study focusing on first-year mortality in incident dialysis patients highlighted a mortality rate of 23.9% within the first 3 months and 19.3% within the first year.

A nationwide study in Sweden explored the causes of hospital admissions and readmissions among patients undergoing hemodialysis and PD. It found a high hospitalization burden, with cardiovascular (CV) events and infections being the most frequent causes of admission. No significant differences in readmission risks between dialysis modalities were observed, but a pattern of readmissions attributed to complications from infections and their interplay with CV diseases was identified [3].

Hemodialysis patients may face hospitalization within a 30-day period due to a variety of factors inherent to their condition and treatment regimen. Understanding these factors is critical for healthcare providers looking to reduce risk and improve patient care. The context and importance of research focused on predicting admissions among hemodialysis patients within a 30-day period is rooted in the unique and complex needs of this patient population. Hemodialysis is an important treatment for patients with end-stage renal disease (ESRD), a condition in which the kidneys can no longer perform their necessary functions [4].

Research undertaken by Flythe et al. [5] delved into the efficacy of predictive models and the impact of modifiable risk factors on minimizing hospital readmissions. Their findings reveal that models based on discharge profiles surpass those based on admission information in predicting readmissions within a 30-day period. This underscores the critical role of enhanced medication education and seamless transitions from hospital to home in lowering readmission rates. By examining discharge data, that study pinpointed adjustable risk factors, advocating for intensified medication education and improved transitions to community care as key strategies for reducing readmissions.

Predicting the likelihood of hemodialysis patients being admitted within 30 days offers a forward-looking strategy aimed at enhancing patient care, alleviating healthcare facility pressures, and optimizing cost management. One research effort devised a prediction model for unplanned readmissions within 30 days by transforming medical records into a unified data format (OMOP-CDM) and integrating data on weather conditions to evaluate environmental influences on readmission rates. Meanwhile, another investigation concentrated on the long-term repercussions of readmissions among Medicare beneficiaries within the initial year of undergoing hemodialysis. This study delved into patterns of readmissions and their correlations with mortality, subsequent hospitalizations, and the likelihood of receiving a kidney transplant. While these studies shed light on the elements that influence readmission rates and the long-term consequences for hemodialysis patients, they stop short of directly tackling the challenge of predicting admissions based on monthly blood test results [6,7].

Research on hemodialysis patients predominantly targets risk and mortality prediction. Each session carries potential complications such as hypotension and infections, impacting patient health and healthcare resources. Decaro et al. [8] applied spectral analysis and machine learning, specifically Support Vector Machine (SVM) and Artificial Neural Network (ANN), in order to predict hematocrit and oxygen saturation levels. This approach allows for better assessment of oxygen deficiency and dialysis efficiency, aiming to minimize session-related risks.

Survival rates for hemodialysis patients are lower, prompting studies on mortality risk prediction. Research has examined factors such as age and body mass index (BMI), utilizing machine learning for predictive models. Garcia-Montemayor et al. [9] found Random Forest superior to Logistic Regression in predicting mortality. Radović et al. [10] reported a 94% accuracy rate with SVM. Wong et al. [11] compared several models, identifying the Generalized Additive Model (GAM) as the most effective for predicting mortality and readmission risks. These findings highlight machine learning’s potential in improving patient outcomes through predictive analytics.

Research on predicting hospital readmission for hemodialysis patients is less common. Yeh et al. [12] used data mining for such predictions, and other studies have applied machine learning for both readmission and mortality risks. Early prediction, especially post-blood tests taken within a 30-days period, is vital for improving outcomes, allowing timely clinical interventions.

This study outlines a methodology beginning with K-Nearest Neighbor (KNN) imputation to address missing data, followed by the Synthesized Minority Oversampling Technique (SMOTE) for class balance. It then applies Decision Tree, Bayesian classifiers, and SVM for classifying potential hospital readmissions within a 30-day period among hemodialysis patients. An ensemble learning strategy will integrate the three models to enhance predictive accuracy.

## 2. Preliminary

### 2.1. Hemodialysis Complications

The Clinical Guidelines for Hemodialysis in Taiwan highlight common complications faced by patients, including anemia and CV diseases. One study [4] pinpointed outpatient medication count and cancer comorbidities as predictors for readmissions within 30 days. Low serum albumin levels and hypotension during dialysis were also identified as significant risk factors. Moreover, comorbid conditions, certain biochemical indicators, and dialysis-related factors like central venous catheter use contribute to the likelihood of readmission, underscoring the complexity of managing hemodialysis patient care effectively [13].

In Taiwan, infections, particularly pneumonia and sepsis, are the leading causes of hospital readmissions for end-stage renal disease (ESRD) patients, with CV diseases also contributing significantly. The rising trend in infection-related readmissions from 2010 to 2018 highlights the vulnerability of ESRD patients to infections due to immune dysfunction and dialysis-related complications [14]. Myocardial infarction is notably the primary CV cause for readmissions. This study aims to delve deeper into the link between anemia, CV issues, malnutrition, and readmission risks, emphasizing the need for monitoring and managing these conditions in order to reduce hospital readmissions.

#### 2.1.1. Anemia

Anemia is common among hemodialysis patients due to reduced erythropoietin from kidney failure, reducing red blood cell count and lowering hemoglobin, causing symptoms like fatigue. Monitoring red blood cell and hemoglobin levels, which are crucial for oxygen transport, is essential for diagnosing anemia. Studies indicate that maintaining hemoglobin above 10 g/dL can improve lifespan and reduce mortality, readmissions, and hospital stays, underscoring the importance of managing anemia in these patients [15,16].

#### 2.1.2. CV Disease

In Taiwan, CV diseases are the leading cause of death, especially in hemodialysis patients, whose risk is significantly higher than the general population. Factors contributing to this increased risk include fluid imbalances and conditions such as AH and diabetes. Monitoring indicators like uric acid, cholesterol, blood glucose, and glycated hemoglobin (HbA1c) is crucial for assessing CV risk. For diabetic hemodialysis patients, regular checks of blood glucose and HbA1c levels are vital for managing their condition and minimizing mortality risk [17].

#### 2.1.3. Renal Osteopathy

Renal osteodystrophy, marked by abnormal bone metabolism, leads to conditions such as osteoporosis and fractures, often due to vitamin D issues or kidneys’ failure to activate vitamin D. This disease results from high phosphorus levels in the blood, stimulating excessive parathyroid activity and calcium loss from bones. Blood tests for phosphorus, calcium, and intact parathyroid hormone (PTH) levels help assess the severity. Elevated phosphorus and calcium are linked to higher mortality risks, while high phosphorus and PTH levels correlate with increased readmissions for CV issues and fractures [18].

#### 2.1.4. Nutrition Problem

Nutritional issues in hemodialysis patients, including protein–energy imbalances and nutrient deficiencies, significantly impact health. Albumin levels, often used to assess malnutrition, correlate with mortality risk, with each 1 g/dl decrease raising mortality by 47%. Despite this, albumin’s reliability as a nutritional marker is debated, partly due to the effect of inflammation on albumin levels. In hemodialysis patients, blood urea nitrogen (BUN) and creatinine, typically kidney function indicators, reflect dietary intake and are linked to readmission rates and mortality, highlighting the complex relationship between nutrition and patient outcomes [19,20,21].

#### 2.1.5. Dialysis Infection

Hemodialysis-related infections, such as hepatitis and dialysis access site infections, significantly increase the risk of hospital readmissions and mortality in patients. These patients are more susceptible to infections due to immune system impairments, including malfunctions in lymphocytes and granulocytes, and factors like malnutrition. The diagnosis of viral infections often relies on specific enzyme level tests, and regular monitoring is crucial for early detection. Managing these risks is essential for improving patient outcomes [22].

To evaluate anemia, red blood cells and hemoglobin levels are measured. For CV disease risk, measurements include uric acid, cholesterol, blood glucose, and HbA1c. Renal bone disease assessment involves phosphate, calcium, and PTH levels. Nutritional status is assessed via albumin, BUN, and creatinine levels. Dialysis-related infection detection uses WBC count, glutamic oxaloacetic transaminase (GOT), glutamic pyruvic transaminase (GPT), alkaline phosphatase, and ferritin levels. These criteria provide comprehensive monitoring for hemodialysis complications, summarized in Table 1 for easy reference.

### 2.2. Research on Hemodialysis Prediction

Yeh et al. [12] used data mining to predict hospital readmissions for hemodialysis patients, employing Temporal Abstraction for laboratory data categorization and algorithms like Decision Tree to identify key predictors such as blood albumin and hemoglobin levels. This approach enabled the identification of specific rules associated with increased readmission risks, demonstrating the potential of machine learning in improving patient management and outcomes in hemodialysis care.

Another study identified several predictors for hospital readmissions in hemodialysis patients, emphasizing the significance of specific blood markers and conditions. Notably, lower-than-average blood urea nitrogen, variations in hemoglobin and albumin levels, the presence of DM combined with suboptimal calcium phosphate product, and albumin levels within the lower normal range, were all highlighted as key factors. Blood albumin, in particular, emerges as a crucial indicator for prognosis, demonstrating the value of targeted monitoring in managing patient health and preventing readmissions [11].

Garcia-Montemayor et al. [9] utilized Random Forest and Logistic Regression to predict mortality in hemodialysis patients over various periods post-initiation, revealing that the Random Forest model generally outperformed Logistic Regression. Key predictive factors varied over time, including creatinine, hemoglobin, and BMI, highlighting the dynamic nature of risk factors influencing patient outcomes. They are summarized in Table 2.

### 2.3. Machine Learning

Machine learning algorithms, ranging from Linear Regression to AdaBoost, are trained to classify or predict data. Uddin et al. [23] reviewed multiple algorithms across published studies, finding SVM, Bayesian classification, and Decision Tree to be the most frequently used. This study will focus on these three algorithms for model training, reflecting their common application in disease prediction research.

#### 2.3.1. Decision Trees

Decision trees are supervised learning algorithms in machine learning, primarily used for handling classification problems. Standard algorithms include ID3, C4.5, and CART. The ID3 algorithm was proposed by Quinlan [24]. Typical ID3 uses entropy, a measure of disorder in information theory, as the criterion for splitting. Entropy can be expressed as $p_{i}\log_{2}p_{i}$; the probability $p_{i}$ represents the frequency of occurrence. The preliminary entropy value is calculated as follows:

(1) $$\mathrm{Entropy}=-\overset{l}{\underset{i=1}{\sum }}p_{i}\log_{2}p_{i}$$

The calculation steps are as follows:

1. The initial value of entropy is calculated by Equation (1)
2. Select the feature result, or the information obtained with minimum entropy as the root node of the decision tree
3. Use the minimum entropy value to build the next layer of the decision tree
4. Repeat steps 1–3 until all subtrees are of a single category and the entropy value is 0.

Later, in 1993, Quinlan proposed C4.5, which improved upon the information gain method used in entropy-based classification. He introduced the Gini index as a criterion for feature selection. Decision Tree is a widely used model-building method in many classification problems, such as sensor classification, medical diagnosis, and speech and text recognition [25]. Decision Tree has shown promising results in predicting the occurrence of low blood pressure during hemodialysis [4].

#### 2.3.2. The Naive Bayes Classifier

The Naive Bayes classifier is a machine learning model based on probabilistic models. It relies on Bayes’ theorem, which describes the probability of an event occurring given some known conditions. The formula for Bayes’ theorem is:

(2) $$P(A|B)=\frac{P(B|A)P\left(A\right)}{P\left(B\right)}$$

P(A) is the probability of event A happening; P(B) is the probability of event B happening; P(A|B) is the probability of event A assuming that event B occurs; and P(B|A) is the probability of event B assuming that event A occurs. Event A and event B are both random events, and the probability of event B is not 0.

As a method used to analyze probability through the Bayesian theorem, the Bayesian classifier is a probability model classifier. Different data models will have different training architectures. The typical Bayesian classification architecture is as follows:

1. Gaussian Naive Bayes Classifier: Primarily used when features are continuous variables and the data follow a normal distribution
2. Multinomial Naive Bayes Classifier: Mainly used when features are discrete variables
3. Bernoulli Naive Bayes Classifier: Similar to the multinomial model, but differs in that Bernoulli features are binary.

In Bayesian classification models, it is assumed that all features are independent and, through probabilistic statistics, unknown data categories are determined to achieve classification. In the medical field, various classifiers generally perform similarly. One of the critical factors in choosing which classifier to apply is its explanatory power. Experiments have shown that physicians prefer explanations provided by Bayesian classifiers and 17 Decision Tree classifiers [26].

#### 2.3.3. Support Vector Machine

SVM belongs to supervised learning algorithms in machine learning, mainly used for classification and regression tasks. The concept involves defining the optimal separating hyperplane in order to classify two linearly separable sets of pattern vectors. The hyperplane that maximizes the distance between the nearest data points belonging to different classes is called the optimal hyperplane. SVM can be divided into linear and nonlinear types.

In linear SVM, the distance between the data and the hyperplane is called the hard margin, when the data are entirely linearly separable, meaning they can be perfectly divided into two classes. The formula for the hard margin is as follows:

(3) $$\vec{w}\;*\vec{x_{i}}-b=1\;\mathrm{or}\;\vec{w}\;*\vec{x_{i}}-b=-1,$$

where $\vec{w}$ is the normal vector, $\vec{x_{i}}$ is the support vector, and b is the displacement term.

When the data cannot be separated into two categories, some can cross the interval boundary or even the hyperplane. The interval between the data and the hyperplane is called the soft interval. The soft interval formula is as follows:

(4) $$\left[\frac{1}{n}\overset{n}{\underset{i=1}{\sum }}max\left(0,1-y_{i}\left(\vec{w}\;*\vec{x_{i}}-b\right)\right)\right]+\lambda ∥\vec{w}{∥}^{2},$$

where y is the classification result, and the parameter λ is used to weigh the relationship between increasing the interval size and ensuring that $\vec{x_{i}}$ is on the correct side of the interval. Boser et al. [27] proposed a method to establish a nonlinear classifier by applying kernel techniques to the maximum margin hyperplane. The central concept is to project the data into a high-dimensional space to find the best hyperplane. The formula is as follows:

(5) $$sgn\left(\left[\overset{n}{\underset{i=1}{\sum }}c_{i}y_{i}k\left(\vec{x_{i}},\vec{z}\right)\right]+b\right),$$

where *k* represents the kernel function, $\vec{z}$ is the new point of classification, and $c_{i}$ is a quadratic function subject to linear constraints. In one study predicting mortality rates among hemodialysis patients, the accuracy rate of the predictive model using SVM reached 94.12% [3].

### 2.4. Ensemble Method

The concept of an ensemble method is to systematically combine several models together in the hope of generating a stronger model. The most basic form is the Voting Method, which determines the predicted class label by majority rule. It is further divided into Majority Voting and Weighted Voting: the former involves simple majority voting, while the latter assigns weights to the individual models’ predictions.

#### 2.4.1. Majority Voting

Majority Voting, also known as Hard Voting, is defined such that, if one of the models predicts a probability of a certain class greater than 50%, that class is chosen. If no class receives more than 50% of the votes, a rejection option is given, and the models refrain from making predictions [28].

If there are T models for a binary classification problem and at least T/2+1 models choose the correct class, assuming the outputs of the models are independent and each model has an accuracy of P, each model makes a correct classification with accuracy P. The probability of obtaining at least T/2+1 correct models out of T, according to [29], is

(6) $$P_{mv}=\overset{T}{\underset{k=\left[\frac{T}{2}+1\right]}{\sum }}\left(\frac{T}{k}\right)\;\;\;p^{k}\left(1-p\right){\;}^{T-k}$$

Lam and Suen [30] have proposed:

If *p* > 0.5, then *P_mv_* increases monotonically in *T*, then $\underset{T\to \infty }{\lim}P_{mv}=1$
If *p* < 0.5, then *P_mv_* decreases monotonically in *T*, then $\underset{T\to \infty }{\lim}P_{mv}=0$
If *p* = 0.5, then *P_mv_* = 0.5 for any T

This result is obtained based on the assumption that the models are statistically independent. However, in practice, models are often highly correlated because they are trained on the same problem. Therefore, it is unrealistic to expect the accuracy of majority voting to converge to 1 as the number of individual models increases [28].

#### 2.4.2. Weighted Voting

Weighted Voting, also known as Soft Voting, is where the model outputs are treated as probabilities instead of simply integrating the results. These probabilities are weighted or averaged, and the class with the highest probability is chosen as the final result. Specific weights are assigned to each class for each model. The formula for the combined output C_j_ for each class is as follows:

(7) $$H^{j}\left(x\right)=\overset{T}{\underset{i=1}{\sum }}w_{i}^{j}h_{i}^{j}\left(x\right)$$

Here, $w_{i}^{j}$ serves as the weight of the model *H^j^* classified in the category C_j_.

It should be noted that Weighted Voting is usually used for homogeneous and heterogeneous ensembles; the probabilities generated by different models can usually only be directly compared with careful calibration [28].

Ensemble methods, integrating multiple models, have shown promise in medical predictions; for instance, Majority Voting, combining Stochastic Gradient Descent (SGD), KNN, Random Forest, and Logistic Regression, reached a 90% accuracy rate in heart disease prediction. Similarly, Weighted Voting, using Random Forest, Logistic Regression, and Naive Bayes, achieved accuracy rates of 78.08% and 97.02% for DM and breast cancer, respectively [31,32]. This study will evaluate Majority and Weighted Voting to determine the most effective prediction method.

## 3. Materials and Methods

### 3.1. Dataset

**Figure 1:**
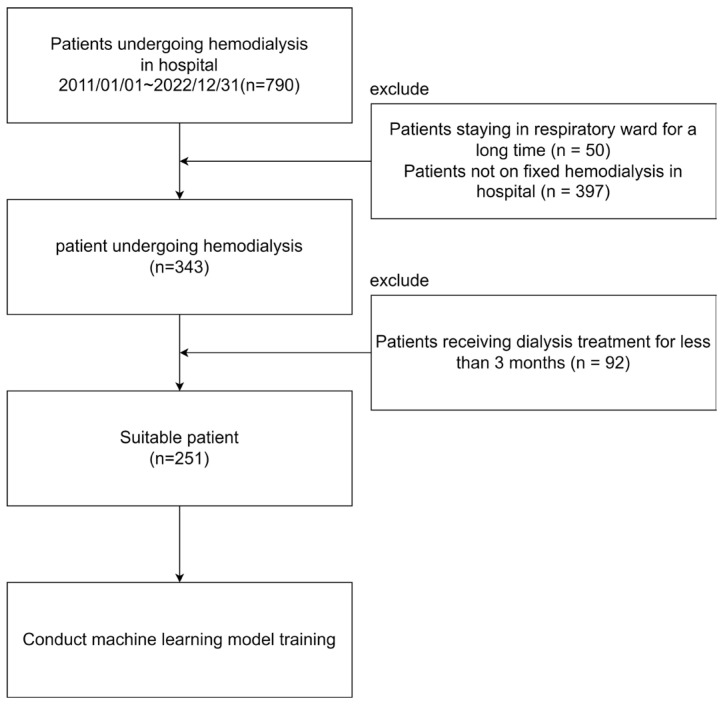
Sample selection.

This study collected data from a Taiwan hospital’s hemodialysis unit between 2011 and 2022. It adhered to the National Kidney Foundation’s testing guidelines. Patients in long-term respiratory care or those not undergoing regular long-term hemodialysis were excluded, focusing on outpatient admissions. Those treated for less than 3 months were also excluded due to incomplete data and emergency conditions. After exclusions, 251 of the initial 790 patients were eligible for analysis, aiming to improve patient care through predictive modeling. Figure 1 illustrates the sample selection steps for this study.

This study analyzed 9367 records from 251 hemodialysis patients, covering basic information, laboratory tests, and hospital readmissions. Anonymized patient IDs (“CHT”) were used for privacy. Monthly tests included routine blood work and specific tests for electrolytes, nutrition, liver function, dialysis efficiency, and lipids. Additional tests like Uric Acid and HbA1c were performed quarterly. Hospital readmission data, indicating readmissions within 30 days post-test, were also analyzed, coded as “1” for no readmissions and “2” for readmissions within this timeframe. Table 3 outlines the dataset, categorizing it into basic patient information, detailed laboratory test results, and hospital readmission data within 30 days post-test.

### 3.2. Research Process

**Figure 2:**
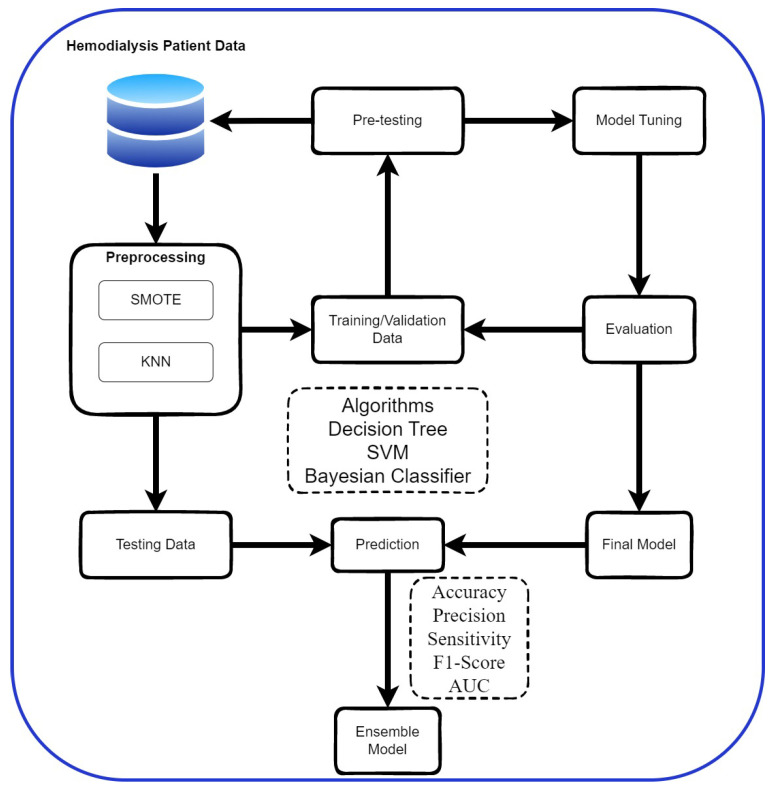
Research process.

This study employs the Python scikit-learn library across five steps: preprocessing data, pre-testing models, training, validating, and creating an ensemble model as shown in Figure 2. Data preprocessing addresses missing values and imbalance. Pre-testing ensures the model meets a 60% accuracy threshold, leading to possible adjustments. Models such as Decision Tree, SVM, and Naive Bayes are trained and evaluated for accuracy. The best model is then chosen for further testing. Finally, an ensemble approach combines three models, aiming for improved accuracy in predicting hospital readmissions.

### 3.3. Data Pre-Processing

Given the clinical origin of the dataset, missing test data are addressed alongside the imbalance between hospital readmissions and non-readmissions, with the latter forming 90.87% of the 9367 records. This imbalance and missing data are critical to model construction, necessitating specific methods for effective handling, which are detailed in the following section.

#### 3.3.1. Data Imbalance Processing

Data imbalance, where one class significantly outnumbers others, can skew model accuracy toward the majority class [33]. Addressing this through oversampling or undersampling is crucial. Studies have proven oversampling, particularly SMOTE, to be more effective than undersampling in balancing datasets [34,35]. This research opts for SMOTE to mitigate data imbalance, enhancing model performance.

#### 3.3.2. Missing Value Handling

Missing values occur from errors in data collection and are addressed by deletion or interpolation, with the latter preferred to avoid data loss. Jadhav et al. [36] evaluated several interpolation methods, finding KNN interpolation most effective. This study adopts KNN for handling missing values, aiming to determine the optimal K value for each model.

### 3.4. Cross-Validation

K-Fold Cross-Validation splits data into K subsets for model testing and validation, using K-1 subsets for training and one for validation, rotating them until each subset has served as the validation set. This method helps assess model generalization [37,38]. The choice of K depends on data bias and measurement error concerns, with K = 10 offering a balance in this study to ensure model accuracy.

### 3.5. Evaluation Metrics

Evaluation metrics classify outcomes into true positive (correctly predicted positive), true negative (correctly predicted negative), false positive (incorrectly predicted positive), and false negative (incorrectly predicted negative). Using these outcomes, metrics such as accuracy, sensitivity, specificity, and AUC (area under the curve) are calculated to assess model performance [39].

Accuracy measures the proportion of true results (both true positives and true negatives) in the total dataset. However, due to potential class imbalance, this might not always be the best performance metric. Precision calculates the accuracy of positive predictions, while sensitivity (or recall) measures the proportion of actual positives correctly identified, which is crucial in medical fields for the minimization of missed positive cases. The F1 score harmonizes precision and sensitivity, and a higher score indicates better model performance. AUC assesses a classifier’s ability across various thresholds, with higher values indicating superior predictive power [22,40]. The formulas for these calculations are presented in Equations (8)–(11):

(8) $$\mathrm{Accuracy}=\left(\frac{\left(\mathrm{TP}+\mathrm{TN}\right)}{\mathrm{Total}}\right)$$

(9) $$Precision=\frac{TP}{TP+FP}$$

(10) $$\mathrm{Sensitivity}=\frac{\mathrm{TP}}{\mathrm{TP}+\mathrm{FN}}$$

(11) $$F1\;\mathrm{Socre}=\frac{2*\left(\mathrm{Precision}\;*\;\mathrm{Recall}\right)}{\left(\mathrm{Precision}\;/\;\mathrm{Recall}\right)}\;\;\;$$

## 4. Result and Discussion

### 4.1. Result

This study will use the Python scikit-learn package for model training, and the modeling process will be presented in the following sections.

#### 4.1.1. Sample Analysis

This study utilized medical records from a hemodialysis unit in a Taiwanese hospital, gathering data on 251 patients differentiated by gender, age, blood type, and medical history, including diabetes, hepatitis, heart disease, high blood pressure, stroke, chronic obstructive pulmonary disease (COPD), and cancer, as listed in Table 4. The data indicate a predominance of elderly patients, aligning with the trend of declining kidney function with age and heightened risk due to chronic conditions and lifestyle factors.

Nearly half of the dialysis patients in Taiwan have diabetes, a major factor in renal function decline. Effective blood glucose monitoring, including HbA1c tests, is critical for managing patient outcomes. Furthermore, comorbidities such as heart disease, AH, and COPD significantly impact these patients, with AH playing a key role in kidney function deterioration. This highlights the importance of regular monitoring and management of blood glucose and blood pressure to prevent complications and hospital readmissions.

While the number of patients with chronic obstructive pulmonary disease (COPD) is relatively low, COPD often co-occurs with conditions such as AH and hyperglycemia. It is noted in clinical observations that heart-related issues such as heart failure, arrhythmia, and myocardial infarction are more prevalent among COPD patients, indicating a significant risk of CV complications alongside COPD [41].

This study’s data, from a hospital in Taiwan, indicates a high occurrence of hepatitis B and C, with specific measures for hemodialysis patients to prevent cross-infection. Cancer history is rare among patients, primarily impacting the kidneys due to urinary system cancers, surgical removal, or chemotherapy toxicity. The records generically note “cancer”, necessitating more detailed data on cancer types.

From the 9367 records of 251 patients, Table 5 displays descriptive statistics such as mean and standard deviation for each variable. Certain tests, as highlighted, have over 50% missing data due to their periodic nature, impacting their availability in the dataset. Clinicians consider these variables significant for assessing the risk of hospital readmissions.

**Figure 3:**
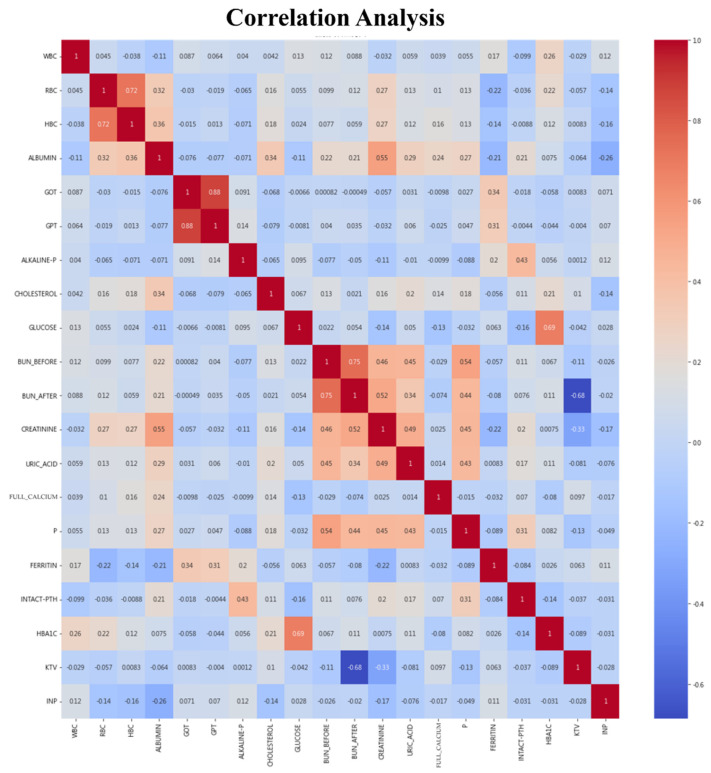
Heatmap of variables correlation analysis.

The heatmap in this study, as shown in Figure 3, illustrates variable correlations, with dark red showing positive and blue indicating negative relationships. Key insights include the following: links between anemia and both RBC and HBC, due to iron’s role in hemoglobin; GOT and GPT signaling liver health, with higher levels indicating liver inflammation; BUN_BEFORE and BUN_AFTER’s reflection of dialysis efficacy, with high values suggesting suboptimal dialysis; the relationship between ALBUMIN, CREATININE, and malnutrition, impacting patient survival; and the connection between phosphorus, dialysis quality, and mortality risk. Additionally, HBA1C and GLUCOSE levels are crucial for managing DM in dialysis patients, highlighting CV disease risks.

On the other hand, BUN_AFTER and KT/V are significantly inversely correlated because KT/V’s calculation subtracts to obtain BUN_AFTER, indicating dialysis adequacy. The National Kidney Foundation suggests that increasing KT/V to 1.2 reduces mortality by 7% per 0.1 increase; therefore, a KT/V of 1.2 is recommended for optimal patient health, with lower values indicating poor dialysis effectiveness.

#### 4.1.2. Data Preprocessing

The study utilized KNNImputer for missing values, selecting the best K via GridSearchCV, testing odd numbers between 1 and 15. Optimal K values varied by model: 11 for Decision Tree, 1 for SVM, and 9 for Bayesian. SMOTE addressed data imbalance, balancing hospital readmission instances. This process prepared the data for modeling, resolving the issues of missing values and imbalance.

#### 4.1.3. Decision Tree Result

The Decision Tree model’s pre-test showed a 100% rate accuracy on the training set, indicating its strong predictive capability for hospital readmissions; however, the test set accuracy rate fell to 90%. To align the accuracies of both sets, the model was optimized by adjusting the tree’s max_depth and min_samples_leaf using GridSearchCV for parameter tuning. The optimal settings were found to be max_depth 14 and min_samples_leaf 13. Final training incorporated evaluation metrics such as accuracy and AUC, with 10% cross-validation ensuring reliable indicator values.

The Decision Tree model’s optimization led to a balance between training and test set performance, with metrics such as accuracy, precision, and AUC closely aligned at 0.92 and 0.91, respectively. This balance indicates enhanced generalization capability and consistency in model performance. The outcomes for both sets are depicted in detail in Table 6.

**Figure 4:**
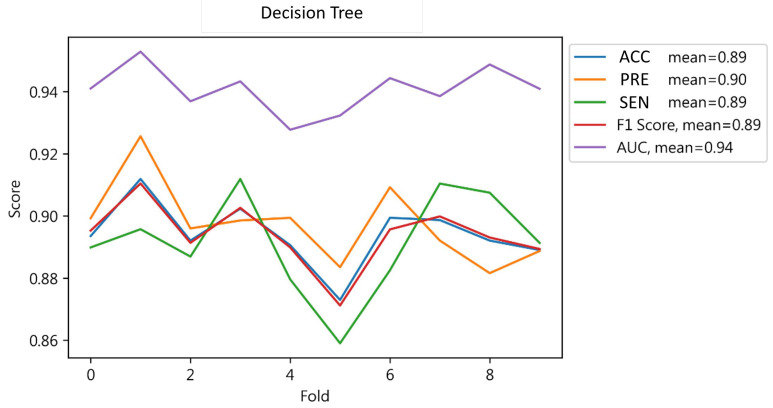
Cross-validation results of Decision Tree.

The model demonstrated strong performance across key metrics in 10-fold cross-validation, with average values of accuracy, sensitivity, F1-score, and AUC indicating its robustness. This underscores the model’s capability to accurately classify instances and maintain a high recall rate with minimal errors, as detailed in Figure 4, where PRE stands for precision and SEN stands for sensitivity; AUC stands for area under curve.

**Figure 5:**
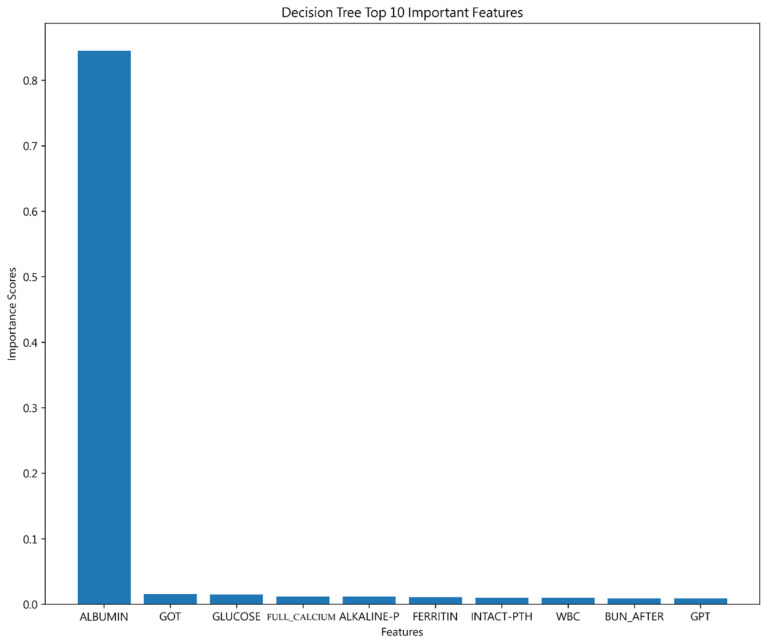
Top 10 features from Decision Tree.

The analysis identified ALBUMIN as the most influential factor in prediction, aligning with earlier discussions of its significance. This underlines the crucial role of ALBUMIN levels in assessing patient outcomes, as detailed in the rankings of influential variables shown in Figure 5.

#### 4.1.4. Support Vector Machine Results

The preliminary test outcomes for both the training and test sets exhibited an accuracy of 0.82 and 0.80, respectively, surpassing the 60% accuracy threshold. Despite achieving over 0.8 accuracy, these results did not outperform the Decision Tree model, prompting further optimization in order to enhance accuracy.

The model’s optimization involved using GridSearchCV to adjust the penalty coefficient C and gamma parameter. Testing C values of 1, 10, 100, and 1000, and gamma values of 1, 0.1, 0.01, 0.001, and 0.0001, the optimal parameters were found to be C at 10 and gamma at 1.

The optimized SVM model demonstrated perfect scores in the training set and strong performance in the test set, with overall metrics indicating a more balanced outcome. Specifically, the differentiation in model accuracy and sensitivity across binary classifications highlights its nuanced predictive capability. The F1-score, as a combined measure of precision and sensitivity, underscores the model’s balanced performance, showcasing its ability to accurately predict outcomes. Further details are presented in Table 7.

**Figure 6:**
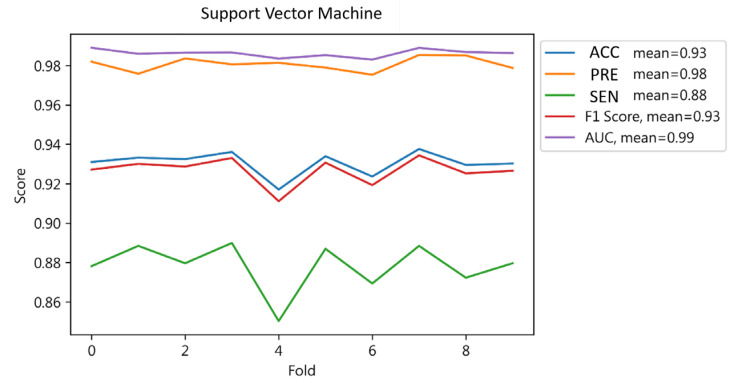
Cross-validation results of SVM.

The cross-validation results (Figure 6) showed an average accuracy of 0.93, an average precision of 0.98, an average sensitivity of 0.88, an average F1-score of 0.93, and an average AUC of 0.99. Cross-validation revealed that the model has a high overall performance with mean scores indicating strong predictive capabilities for hospital readmissions. Notably, high average accuracy and AUC reflect the model’s effectiveness in differentiating cases, while the sensitivity score underscores its precision in prediction. This demonstratesVM SVM’s potential in accurately forecasting patient readmissions.

**Figure 7:**
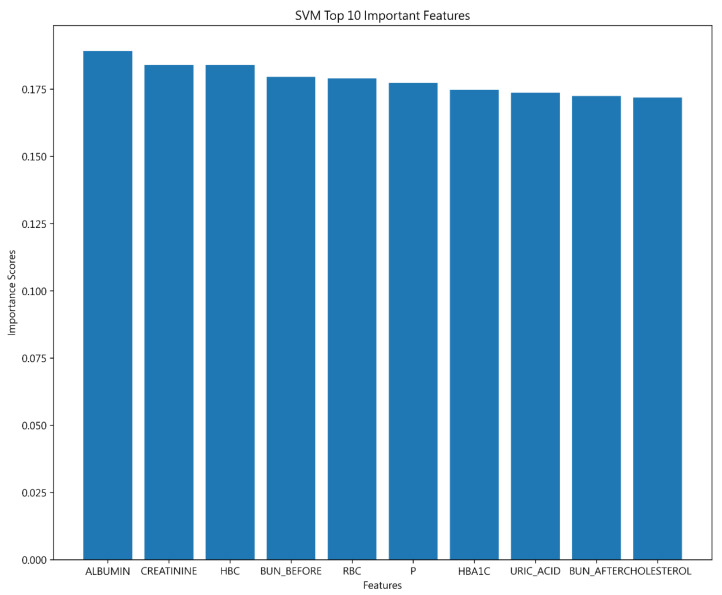
Top 10 features of SVM.

This study identified the most impactful variables for hospital readmission predictions, with ALBUMIN, CREATININE, and HBC leading. In alignment with Decision Tree findings, ALBUMIN was proven to be crucial. The close scoring among the top 10 variables suggests that each significantly affects model performance, as detailed in Figure 7.

#### 4.1.5. Bayesian Classifier Results

The Bayesian classifier’s unique structure limits parameter adjustments, affecting its performance. Results show that it has a lower prediction efficiency compared to Decision Tree and SVM models. It is weaker in negative case predictions but its strengths lie in sensitivity and F1-score for positive classifications. This leads to an less effective prediction outcome overall, as detailed in Table 8.

**Figure 8:**
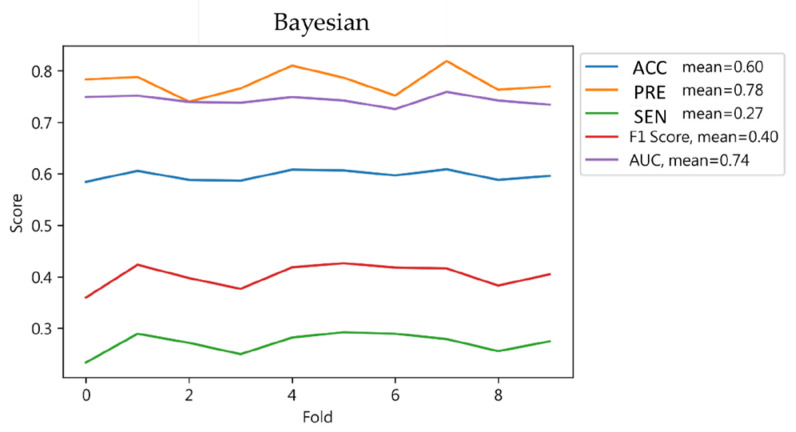
Cross-validation results of Bayesian classification.

The model’s 10-fold cross-validation showed an average accuracy and relatively high sensitivity, indicating a decent overall prediction ability but exposing limitations in accurately identifying negative cases. The lower F1-score and AUC values suggest a need for improvement in model performance, especially in specificity and precision, as detailed in Figure 8.

**Figure 9:**
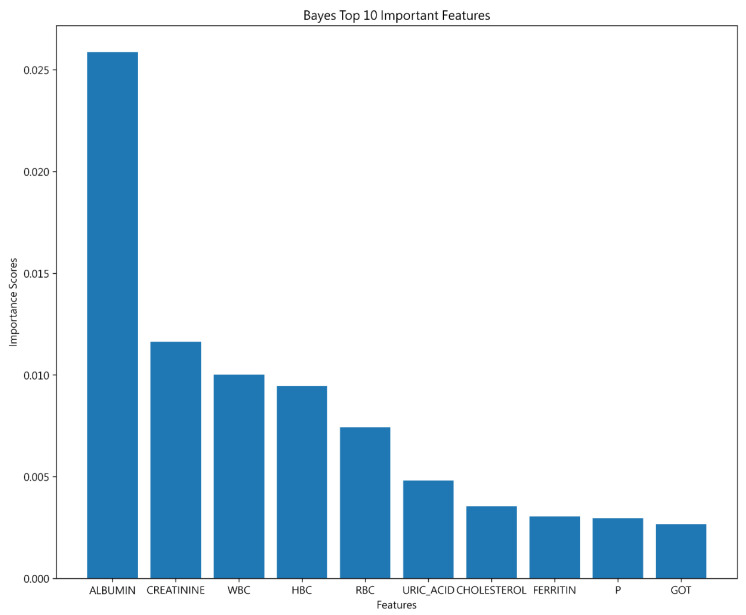
Top 10 features of Bayesian.

In the Bayesian classification model, ALBUMIN emerged as the most pivotal variable for prediction, mirroring findings from Decision Tree and SVM. CREATININE and WBC also significantly influenced outcomes, although their importance varied across models. Unlike Decision Tree, the distinction in feature impact was less pronounced, indicating a more uniform influence on the Bayesian model, with ALBUMIN notably dominating. The ranking of top variables is displayed in Figure 9 for clarity.

#### 4.1.6. Result of Ensemble Learning

This study opted for the voting algorithm for ensemble learning model training. The Majority Voting results demonstrated a training set performance of 0.96 across accuracy, precision, sensitivity, F1-score, and AUC metrics. The test set showed slightly lower results at 0.92 for these metrics.

The study determined the optimal weights for the Weighted Voting method as 1:2:1, achieving an average accuracy rate of 96%, as depicted in a specific figure. With these weights, both training and testing phases reached a 0.96 score across all metrics, including accuracy, precision, sensitivity, and F1-score for Weighted Voting, as detailed in Table 9.

**Figure 10:**
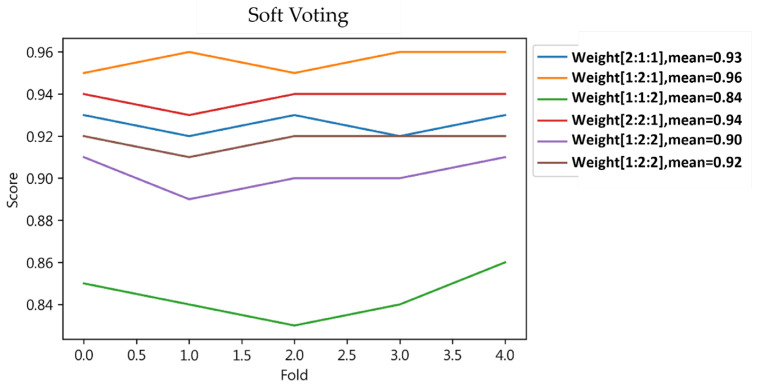
Soft Voting weighted.

Weighted Voting yielded better outcomes than Majority Voting. While Majority and Weighted Voting showed different results in training, Weighted Voting’s comprehensive evaluation through the F1-score revealed a more balanced performance, effectively predicting both positive and negative cases, detailed in Table 9 and Figure 10.

### 4.2. Discussion

#### 4.2.1. Machine Learning and Ensemble Learning

This study evaluated three machine learning models for predicting hemodialysis patient readmissions, summarized in Table 10. SVM emerged as the most effective model, closely followed by Decision Tree, which also demonstrated high performance. This indicates the strong capability of these models in classifying data within the feature space.

The Bayes classifier’s performance was found to be lacking, particularly in distinguishing between negative and positive cases, as highlighted by its sensitivity and F1-scores. This challenge arises partly because Bayes classifiers treat features as independent, which is a problematic assumption when dealing with complex, interrelated features. The presence of variable correlations further complicates classification. This underscores the necessity of exploring multiple classifiers and potential of combining their strengths for more accurate predictions.

This study employed ensemble methods alongside individual models to enhance prediction accuracy, utilizing both Majority and Weighted voting techniques. Majority Voting aggregates predictions by majority rule, but only its accuracy paralleled that of the SVM model. Conversely, Weighted Voting, by adjusting SVM’s weight, yielded superior results, highlighting ensemble learning’s potential for improving accuracy.

Ensemble learning works better than a single specific machine learning model in many cases because, while a single model tends to overfit the training data, ensemble learning can reduce this risk by integrating the predictions of multiple models. Ensemble learning often generalizes better to unseen data because it combines the predictions of multiple models, resulting in more robust and generalized predictive capabilities. A single model may be affected by noise and outliers; however, by integrating multiple models, ensemble learning can reduce this effect because outliers are likely to affect only one or a few of the models. Since ensemble learning uses multiple models, it is more robust and, even if one of the models performs poorly, the performance of the overall ensemble model can still be maintained.

Ensemble learning is highly valuable for clinical use, offering insights for medical decisions and more accurate patient risk assessments. By merging multiple models’ predictions, it addresses single model limitations and enhances accuracy for clinical relevance. This study indicates that Weighed Voting methods yield optimal predictions for hemodialysis patient readmissions, serving as a significant tool for clinical decisions and showcasing the benefits of model combination in tackling clinical challenges.

#### 4.2.2. Important Features of 30-Day Hospital Admissions for Hemodialysis Patients

The pivotal features from the three models, as detailed in Section 4.1, are collectively presented in Table 11, offering a comprehensive overview of key predictors for hospital readmissions among hemodialysis patients. The characteristics of these important variables are explained in the following sections.

ALBUMIN: Albumin is crucial for predicting hospital readmissions within a 30-day period among maintenance hemodialysis patients due to its role as a biomarker for nutritional status and inflammation. Low levels of albumin indicate malnutrition and/or chronic inflammation, which are associated with increased risks of complications that lead to hospital readmission. Therefore, albumin levels offer valuable insight into patients’ health and the effectiveness of their dialysis treatment, guiding clinical decisions towards potentially reducing readmission rates.

CREATININE: Creatinine is a key feature for predicting hospital readmissions within a 30-day period among maintenance hemodialysis patients because it reflects kidney function and dialysis efficacy. Elevated creatinine levels may indicate inadequate dialysis or worsening kidney function, which can lead to complications requiring hospital readmission. Monitoring creatinine helps assess the adequacy of dialysis treatment and patients’ overall health status, making it a vital marker for predicting hospital readmission risk.

HBC: Hemoglobin concentration is a crucial feature for predicting hospital readmissions within a 30-day period among maintenance hemodialysis patients because it directly relates to patients’ anemia status. Anemia is a common condition in hemodialysis patients, impacting their overall health and increasing hospital readmission risks. Low HBC levels indicate poor anemia management, which can lead to complications requiring hospital care. Therefore, monitoring HBC can provide valuable insights into patients’ health status and predict readmission risks.

The contribution of blood test data to hospital readmissions is mainly reflected in the following aspects: Blood tests can be used to monitor the control of chronic diseases such as diabetes, high blood pressure, heart disease, and more. By regularly checking blood markers, healthcare workers can detect and manage illnesses promptly, reducing prehospitalization risk. Blood tests can provide important information about an individual’s health status, such as blood sugar levels, cholesterol levels, white blood cell counts, and more. These measures can assess an individual’s overall health and may be related to readmission risk. Blood test data can provide vital information for assessing and managing readmission risk, helping to improve the quality of patient care, reduce medical costs, and improve overall medical outcomes.

Taiwan’s medical insurance system is worthy of note within the global context. The hemodialysis room performs blood tests for hemodialysis patients every month. In addition to allowing kidney patients to understand their physiological status, regular blood work can also prevent the occurrence of dialysis complications early. However, studies have not yet been undertaken that specifically focus on analyzing monthly blood tests to predict hospitalizations within a 30-day period among maintenance hemodialysis patients. Typically, studies have either focused on different aspects of hemodialysis patient care such as long-term outcomes and readmission patterns, rather than directly analyzing monthly blood test data to predict admissions.

The contribution of this study in analyzing monthly blood tests to predict hospitalizations within a 30-day period among maintenance hemodialysis patients is significant and multifaceted, especially in the context of the Taiwanese healthcare system. These contributions include the following:

1. This study addresses a specific research gap by using monthly blood test data to predict short-term hospitalizations in hemodialysis patients. This area has previously been neglected in favor of long-term outcomes and general readmission trends.
2. By analyzing routine blood tests to predict the likelihood of hospitalization within 30 days, healthcare providers can identify high-risk patients earlier. This enables the implementation of preventive measures to avoid hospitalization and potential complications associated with hemodialysis.
3. Predictive analytics can help healthcare organizations better allocate resources by identifying patients at higher risk of hospitalization, ensuring interventions are directed where they are needed most.
4. Early detection and prevention of potential complications can significantly improve the quality of life of hemodialysis patients and reduce the number of hospitalizations, allowing them to maintain a more stable and comfortable daily life.
5. Insights from this study can inform healthcare policies and strategies, especially in improving the efficiency and effectiveness of Taiwan’s renowned health insurance system. It can serve as a model for integrating predictive analytics into daily patient care and has the potential to be adopted in similar healthcare settings around the world.
6. This study contributes to the wider field of nephrology by providing a new approach to managing hemodialysis patients through the strategic use of routine clinical data, setting a precedent for future research and practice.

Overall, this study represents a major advance in the proactive management of hemodialysis patients, leveraging routine medical data to improve patient outcomes and healthcare efficiency.

## 5. Conclusions

Historically, hemodialysis studies predominantly aimed at predicting mortality, with less emphasis on forecasting hospital readmissions due to worsening health conditions in patients. Nonetheless, accurately predicting hospital readmission risks could significantly enhance patient survival rates. This study is the first to use monthly blood test data from hemodialysis patients to predict hospital admissions within a 30-day period. The initial step involved using the K-Nearest Neighbor method for imputation of missing data, followed by employing the Synthetic Minority Oversampling Technique (SMOTE) to tackle the challenge of data imbalance. Subsequent analyses utilized machine learning algorithms to predict the risk of hospital readmissions within a 30-day period for hemodialysis patients. Among the tested machine learning models, Support Vector Machine (SVM) showed the highest initial accuracy, achieving a 93% rate. The incorporation of ensemble learning methods further enhanced model performance, boosting accuracy rates to 96%. These findings underscore the potential of ensemble learning models to leverage monthly blood test data effectively for predicting short-term hospital readmission risks among hemodialysis patients. These advancements hold significant implications for the field of precision medicine. This study can also serve as a foundational step towards more personalized and effective healthcare solutions for hemodialysis patients.

In addition, this study emphasizes the liver index ALBUMIN as a key predictor across models, particularly highlighting its clinical significance beyond malnutrition, which is often linked to inflammation. The kidney function indicator CREATININE reflects muscle mass and dialysis efficacy, marking its importance alongside ALBUMIN in readmission risk. Additionally, HBC and WBC are identified as critical for assessing anemia and infection risk, respectively. These insights offer clinicians valuable indicators for evaluating hemodialysis patients’ readmission risk, underscoring the importance of these variables in clinical assessments.

### 5.1. Limitations

This study offers initial insights into predicting hemodialysis patient readmissions, acknowledging limitations such as regional data scope and a focus on blood tests without considering demographics or medical history. Future research could broaden variables, apply different ensemble methods or deep learning, and expand sample sizes to enhance prediction accuracy and reliability. Extending the prediction timeline and exploring practical applications in clinical settings could also provide valuable improvements, aiming for broader applicability as well as refinement based on clinical feedback.

### 5.2. Implications

Predicting hospital readmissions within a 30-day period among maintenance hemodialysis patients, using monthly blood test data, can significantly impact patient care and healthcare resource management. This approach allows for early identification of at-risk patients, enabling proactive intervention that can reduce readmissions, enhance patient outcomes, and optimize healthcare expenditures. By analyzing blood test data, healthcare providers can detect underlying health issues sooner, tailor treatments more effectively, and improve the overall quality of care for hemodialysis patients.

In addition, using machine learning to predict hospital readmissions within a 30-day period among maintenance hemodialysis patients has profound implications. It enables early identification of individuals at risk, improving patient management and potentially reducing readmission rates. This approach can lead to more personalized care plans, optimizing treatment efficacy and patient outcomes. Additionally, it may offer significant cost savings for healthcare systems by minimizing unnecessary hospitalizations, thus allocating resources more efficiently and enhancing the overall quality of care for hemodialysis patients.

## Figures and Tables

**Table 1:** Complications and values to be monitored.

Complication	Monitoring Value
Anemia	Erythrocyte, hemoglobin
CV disease	Uric acid, cholesterol, blood sugar, glycated hemoglobin
Renal osteopathy	Phosphorus ion, calcium ion, complete parathyroid hormone
Dialysis infection	White blood cells, transaminase glutamine phenylacetic acid, transaminase glutamine pyruvate, basic phosphoric acid, serum ferritin
Nutrition problem	Blood protein, urea nitrogen, creatinine

**Table 2:** Related research on hemodialysis prediction.

Subject	Method	Author
Hospital readmissions status	Decision Tree, MSApriori	Yeh et al. [12]
Hospital readmissions risk	GLM (Generalized Linear Model), GAM (Generalized Additive Model), Classification Tree, Random Forest	Wong et al. [11]
Hemodialysis patient mortality	Random Forest, Logistic Regression	Garcia-Montemayor et al. [9]

**Table 3:** Dataset description.

	Variable Name	Variable Description	Attributes
Basic information	CHT	Anonymous ID of patient	nonmetric
Inspection data	WBC (white blood cell)	White blood cell count	metric
	RBC (red blood cell)	Red blood cell count	metric
	HbC	Hemoglobin	metric
	ALBUMIN	Albumin	metric
	GOT (glutamic oxaloacetic transaminase)	Serum glutamate phenylacetate transaminase	metric
	GPT (glutamic pyruvic transaminase)	Serum glutamate-pyruvate transaminase	metric
	ALKALINE-P	Alkaline phosphoric acid	metric
	CHOLESTEROL	Total cholesterol	metric
	GLUCOSE	blood sugar	metric
	BUN_BEFORE	Blood urea nitrogen before dialysis	metric
	BUN_AFTER	Blood urea nitrogen after dialysis	metric
	CREATININE	Creatinine	metric
	URIC_ACID	Uric acid	metric
	FULL_CALCIUM	Calcium ions	metric
	P	Phosphorus ions	metric
	FERRITIN	Ferritin	metric
	INTACT-PTH	Parathyroxine immunoassay	metric
	HBA1C	Glycated hemoglobin	metric
	KTV(Kt/V)	Urea nitrogen dialysis efficiency	metric
Hospital readmissions	INP	Hospitalized within 30 days	non-metric

**Table 4:** Descriptive statistics of patients.

Character	Data (*n* = 251)
Sex	Male (121), female (130)
Age	Over 70 years old (139), under 70 years old (112)
Blood type	A (47), B (73), O (118), AB (13)
History of diabetes	Y (121), N (130)
History of hepatitis B	Y (18), N (233)
History of hepatitis C	Y (65), N (186)
Cardiac history	Y (72), N (179)
History of AH	Y (175), N (76)
History of stroke	Y (211), N (40)
History of COPD	Y (20), N (231)
History of cancer	Y (3), N (248)

**Table 5:** Summary of data.

	MEAN	STD	MAX	MIN
WBC (white blood cell)	6.84	2.43	34.5	0.75
RBC (red blood cell)	3.42	0.56	6.08	1.58
HbC	10.19	1.40	16	4.9
ALBUMIN	3.86	0.45	5.3	1.6
GOT (glutamic oxaloacetic transaminase)	19.23	59.45	5121	2
GPT (glutamic pyruvic transaminase)	16.02	30.78	1976	2
ALKALINE-P	93.70	52.83	918	24
CHOLESTEROL	157.69	39.96	437	63
GLUCOSE	197.87	109.7	1034	29
BUN_BEFORE	67.01	19.97	259	6
BUN_AFTER	16.40	6.91	72	2
CREATININE	9.07	2.56	22.82	1.13
URIC_ACID	6.68	1.60	16.9	0.9
TOTAL_CALCIUM	9.16	0.89	13.5	3.1
P	4.99	1.59	15	0.4
FERRITIN	397.44	446.38	9570.25	4.7
INTACT-PTH	442.41	441.21	4394.6	3.9
HBA1C (glycated hemoglobin)	7.03	2.03	16.6	3.1
KTV(Kt/V)	1.46	0.29	3.71	0.42

**Table 6:** Decision Tree results.

		ACC	PRE	SEN	F1-Score	Volumn
Training	0		0.93	0.92	0.92	6810
	1		0.92	0.93	0.92	6809
	Result	0.92	0.92	0.92	0.92	
Testing	0		0.91	0.90	0.90	1702
	1		0.90	0.91	0.91	1703
	Result	0.91	0.91	0.91	0.91	

PRE stands for precision and SEN stands for sensitivity.

**Table 7:** Results of SVM.

		ACC	PRE	SEN	F1-Score	Volumn
Training	0		1.0	1.0	1.0	6810
	1		1.0	1.0	1.0	6809
	Result	1.0	1.0	1.0	1.0	
Testing	0		0.90	0.98	0.94	1702
	1		0.98	0.89	0.93	1703
	Result	0.93	0.94	0.93	0.93	

PRE stands for precision and SEN stands for sensitivity.

**Table 8:** Results of Bayesian classifier.

		ACC	PRE	SEN	F1-Score	Volumn
Training	0		0.56	0.92	0.70	6809
	1		0.78	0.27	0.40	6810
	Result	0.60	0.67	0.60	0.55	
Testing	0		0.57	0.92	0.70	1703
	1		0.79	0.29	0.43	1702
	Result	0.61	0.68	0.61	0.56	

PRE stands for precision and SEN stands for sensitivity.

**Table 9:** Ensemble learning results.

		Training				Testing			
		ACC	PRE	SEN	F1 Score	ACC	PRE	SEN	F1 Score
Hard Voting	0		0.94	0.98	0.96		0.88	0.97	0.92
	1		0.98	0.94	0.96		0.96	0.86	0.91
	Result	0.96	0.96	0.96	0.96	0.92	0.92	0.92	0.92
Soft Voting	0		1.0	1.0	1.0		0.93	0.99	0.96
	1		1.0	1.0	1.0		0.99	0.93	0.96
	Result	1.0 *	1.0 *	1.0 *	1.0 *	0.96 *	0.96 *	0.96 *	0.96 *

* PRE stands for precision and SEN stands for sensitivity.

**Table 10:** Comparison results of the models.

	Testing				
	ACC	PRE	SEN	F1 Score	AUC
DT	0.91	0.91	0.91	0.91	0.91
SVM	0.93	0.94	0.93	0.93	0.93
Bayesian	0.61	0.68	0.61	0.56	0.61
Ensemble	0.96 *	0.96 *	0.96 *	0.96 *	-

* PRE stands for precision and SEN stands for sensitivity.

**Table 11:** Important blood features of the three models.

	Decision Tree	SVM	Bayesian
Important features	ALBUMIN	ALBUMIN	ALBUMIN
		CREATININE	CREATININE
		HBC	WBC

## Data Availability

Dataset available on request from the authors.

## References

[1] Himmelfarb J., Vanholder R., Mehrotra R., Tonelli M. (2020). The current and future landscape of dialysis. Nat. Rev. Nephrol..

[2] Heaf J., Heiro M., Petersons A., Vernere B., Povlsen J.V., Sørensen A.B., Clyne N., Bumblyte I., Zilinskiene A., Randers E. (2022). First-year mortality in incident dialysis patients: Results of the Peridialysis study. BMC Nephrol..

[3] Xu Y., Li L., Evans M., Xu H., Lindholm B., Carrero J.J. (2021). Burden and causes of hospital admissions and readmissions in patients undergoing hemodialysis and peritoneal dialysis: A nationwide study. J. Nephrol..

[4] Gómez-Pulido J.A., Gómez-Pulido J.M., Rodríguez-Puyol D., Polo-Luque M.L., Vargas-Lombardo M. (2021). Predicting the appearance of hypotension during hemodialysis sessions using machine learning classifiers. Int. J. Environ. Res. Public Health.

[5] Flythe J.E., Katsanos S.L., Hu Y., Kshirsagar A.V., Falk R.J., Moore C.R. (2016). Predictors of 30-Day Hospital Readmission among Maintenance Hemodialysis Patients: A Hospital’s Perspective. Clin. J. Am. Soc. Nephrol..

[6] Ryu B., Yoo S., Kim S., Choi J. (2021). Thirty-day hospital readmission prediction model based on common data model with weather and air quality data. Sci. Rep..

[7] Ross K.H., Jaar B.G., Lea J.P., Masud T., Patzer R.E., Plantinga L.C. (2019). Long-term outcomes among Medicare patients readmitted in the first year of hemodialysis: A retrospective cohort study. BMC Nephrol..

[8] Decaro C., Montanari G.B., Molinari R., Gilberti A., Bagnoli D., Bianconi M., Bellanca G. (2019). Machine learning approach for prediction of hematic parameters in hemodialysis patients. IEEE J. Transl. Eng. Health Med..

[9] Garcia-Montemayor V., Martin-Malo A., Barbieri C., Bellocchio F., Soriano S., de Mier V.P.-R., Molina I.R., Aljama P., Rodriguez M. (2021). Predicting mortality in hemodialysis patients using machine learning analysis. Clin. Kidney J..

[10] Radović N., Prelević V., Erceg M., Antunović T. (2022). Machine learning approach in mortality rate prediction for hemodialysis patients. Comput. Methods Biomech. Biomed. Eng..

[11] Wong M.M., Thijssen S., Wang Y., Usvyat L.A., Xiao Q., Kotanko P., Maddux F.W. (2020). Prediction of mortality and hospital readmissions risk using nutritional indicators and their changes over time in a large prevalent hemodialysis cohort. J. Ren. Nutr..

[12] Yeh J.Y., Wu T.H., Tsao C.W. (2011). Using data mining techniques to predict hospital readmissions of hemodialysis patients. Decis. Support Syst..

[13] Assimon M.M., Flythe J.E. (2017). Thirty-day hospital readmissions in the hemodialysis population: A problem well put, but half-solved. Clin. J. Am. Soc. Nephrol..

[14] Lee C.C., Hsu C.C., Lin M.H., Chen K.H., Wu I.W. (2022). Hospital readmissions in patients with dialysis in Taiwan: A nationwide population-based observational study. J. Formos. Med. Assoc..

[15] Ma J.Z., Ebben J., Xia H., Collins A.J. (1999). Hematocrit level and associated mortality in hemodialysis patients. J. Am. Soc. Nephrol..

[16] Ofsthun N., Labrecque J., Lacson E., Keen M., Lazarus J.M. (2003). The effects of higher hemoglobin levels on mortality and hospital readmissions in hemodialysis patients. Kidney Int..

[17] Hill C.J., Maxwell A.P., Cardwell C.R., Freedman B.I., Tonelli M., Emoto M., Inaba M., Hayashino Y., Fukuhara S., Okada T. (2014). Glycated hemoglobin and risk of death in diabetic patients treated with hemodialysis: A meta-analysis. Am. J. Kidney Dis..

[18] Block G.A., Klassen P.S., Lazarus J.M., Ofsthun N., Lowrie E.G., Chertow G.M. (2004). Mineral metabolism, mortality, and morbidity in maintenance hemodialysis. J. Am. Soc. Nephrol..

[19] de Mutsert R., Grootendorst D.C., Indemans F., Boeschoten E.W., Krediet R.T., Dekker F.W., Netherlands Cooperative Study on the Adequacy of Dialysis-II Study Group (2009). Association between serum albumin and mortality in dialysis patients is partly explained by inflammation, and not by malnutrition. J. Ren. Nutr..

[20] Lowrie E.G., Laird N.M., Parker T.F., Sargent J.A. (1981). Effect of the hemodialysis prescription on patient morbidity: Report from the National Cooperative Dialysis Study. N. Engl. J. Med..

[21] Walther C.P., Carter C.W., Low C.L., Williams P., Rifkin D.E., Steiner R.W., Ix J.H. (2012). Interdialytic creatinine change versus predialysis creatinine as indicators of nutritional status in maintenance hemodialysis. Nephrol. Dial. Transplant..

[22] Daugirdas J.T., Blake P.G., Ing T.S. (2007). Handbook of Dialysis.

[23] Uddin S., Khan A., Hossain M.E., Moni M.A. (2019). Comparing different supervised machine learning algorithms for disease prediction. BMC Med. Inform. Decis. Mak..

[24] Quinlan J.R. (1986). Induction of decision trees. Mach. Learn..

[25] Arifuzzaman M., Hasan M.R., Toma T.J., Hassan S.B., Paul A.K. (2023). An Advanced Decision Tree-Based Deep Neural Network in Nonlinear Data Classification. Technologies.

[26] Kononenko I. (2001). Machine learning for medical diagnosis: History, state of the art and perspective. Artif. Intell. Med..

[27] Boser B.E., Guyon I.M., Vapnik V.N. A training algorithm for optimal margin classifiers. Proceedings of the Fifth Annual Workshop on Computational Learning Theory.

[28] Zhou Z.H. (2012). Ensemble Methods: Foundations and Algorithms.

[29] Hansen L.K., Salamon P. (1990). Neural network ensembles. IEEE Trans. Pattern Anal. Mach. Intell..

[30] Lam L., Suen S.Y. (1997). Application of majority voting to pattern recognition: An analysis of its behavior and performance. IEEE Trans. Syst. Man Cybern.-Part A Syst. Hum..

[31] Atallah R., Al-Mousa A. Heart disease detection using machine learning majority voting ensemble method. Proceedings of the 2019 2nd International Conference on New Trends in Computing Sciences (Ictcs).

[32] Kumari S., Kumar D., Mittal M. (2021). An ensemble approach for classification and prediction of diabetes mellitus using soft voting classifier. Int. J. Cogn. Comput. Eng..

[33] He H., Garcia E.A. (2009). Learning from imbalanced data. IEEE Trans. Knowl. Data Eng..

[34] Fotouhi S., Asadi S., Kattan M.W. (2019). A comprehensive data level analysis for cancer diagnosis on imbalanced data. J. Biomed. Informatics.

[35] Paing M.P., Pintavirooj C., Tungjitkusolmun S., Choomchuay S., Hamamoto K. Comparison of sampling methods for imbalanced data classification in random forest. Proceedings of the 2018 11th Biomedical Engineering International Conference (BMEiCON).

[36] (2019). Chiang Mai, Thailand Comparison of performance of data imputation methods for numeric dataset. Appl. Artif. Intell..

[37] Hastie T., Tibshirani R., Friedman J.H., Friedman J.H. (2009). The Elements of Statistical Learning: Data Mining, Inference, and Prediction.

[38] Rodriguez J.D., Perez A., Lozano J.A. (2009). Sensitivity analysis of k-fold cross validation in prediction error estimation. IEEE Trans. Pattern Anal. Mach. Intell..

[39] Sokolova M., Lapalme G. (2009). A systematic analysis of performance measures for classification tasks. Inf. Process. Manag..

[40] Hicks S.A., Strümke I., Thambawita V., Hammou M., Riegler M.A., Halvorsen P., Parasa S. (2022). On evaluation metrics for medical applications of artificial intelligence. Sci. Rep..

[41] Sin D.D., Anthonisen N.R., Soriano J.B., Agusti A.G. (2006). Mortality in COPD: Role of comorbidities. Eur. Respir. J..

